# Health-care resource use and costs associated with inflammatory bowel disease in northwest London: a retrospective linked database study

**DOI:** 10.1186/s12876-024-03559-3

**Published:** 2024-12-30

**Authors:** Zia UI-Haq, Luiz Causin, Tahereh Kamalati, Durgesh Kahol, Trishan Vaikunthanathan, Charlotte Wong, Naila Arebi

**Affiliations:** 1 Imperial College Health Partners, London, UK; 2 Janssen-Cilag Ltd, High Wycombe, UK; 3 Department of Inflammatory Bowel Disease, St Mark’s National Bowel Hospital, Central Middlesex Hospital, Acton Lane, London, NW10 7NS UK; 4 Department of Metabolism, Digestion and Reproduction, Imperial College London, London, UK

**Keywords:** Inflammatory bowel disease, Cost, Outcomes, Biologics, Active disease, Remission

## Abstract

**Background:**

With 20–40% of patients who have inflammatory bowel disease (IBD) not responding to therapy, resource use and costs can be high. We performed a descriptive analysis of health-care data for IBD management in the National Health Service to explore potential areas for improvement.

**Methods:**

In this exploratory study, we analysed real-world data from the Discover dataset for adults with a diagnosis of incident IBD recorded in northwest London, UK, between 31 March, 2016, and 31 March, 2020. We compared mean visit numbers and primary and secondary care costs per patient to examine resource use and costs for active disease versus remission.

**Results:**

We included 7,733 patients (5,872 with ulcerative colitis [UC], 1,427 with Crohn’s disease [CD], and 434 with codes for both [termed IBD-undefined in this study]). Remission was recorded in 19,218 (82%) of 23,488 observations for UC, 4,686 (82%) of 5,708 for CD, and 1,122 (65%) for IBD-undefined observations. Health-care resource use was significantly higher with active disease in all settings except primary care for UC. Total health-care costs were greater with active disease than remission for all diagnoses (all *p* < 0.0001). The main driver of costs was inpatient hospital care among those with active disease; elective inpatient costs were high among patients with UC and IBD-undefined in remission.

**Conclusions:**

Higher health-care resource use and costs were observed with active disease, which underscores the importance of early induction and maintenance of remission in UC and CD. Updated strategies that incorporate treat to target may offer cost benefits by the offsetting of biologic drug costs with a reduction in costly inpatient hospital stays.

**Trial registration:**

This trial was not registered as it used pseudonymised retrospective data.

**Supplementary Information:**

The online version contains supplementary material available at 10.1186/s12876-024-03559-3.

## Background

Inflammatory bowel disease (IBD) is an umbrella term for chronic idiopathic inflammatory diseases of the gastrointestinal system, encompassing ulcerative colitis (UC) and Crohn’s disease (CD) [1]. Of the various presentations, a relapsing-remitting inflammatory course is the most common [2, 3]. Progressive disease with complications is associated with hospitalisations, surgery, and impacts on quality of life and work productivity, which can have further psychological, social, and physical consequences [1, 4–6].

IBD places substantial health and economic burdens on communities worldwide. In the USA and Europe, more than 3 million people are estimated to have IBD, whilst in the UK, the prevalence is around 0.5‒0.8%.^7^–^11^ Owing to factors such as early onset, no cure, and low mortality, IBD is going through a period of compounding prevalence. Thus, in high-income countries, prevalence is estimated to reach 1% by 2030 [12]. Furthermore, with the disease exhibiting a bimodal age distribution and in the context of an ageing general population, older people now represent the largest-growing population of patients living with IBD. This will pose unique challenges and considerations to health-care professionals in terms of diagnosis and treatment decisions [13–15].

Health-care use in IBD, particularly during active stages, incurs direct and indirect costs to health-care systems, patients, their families, and society as a whole [16, 17]. Direct health-care costs are attributed to diagnostic delays, disease activity, drug and surgical treatments, and monitoring for treatment response and complications, such as infections. Furthermore, although a broad range of therapies is available, ranging from 5-aminosalicylates (for UC), corticosteroids, immunomodulators, small molecules, and biologic agents [18], up to 20–40% of patients experience non-response, loss of response, and intolerance to therapy [19, 20]. The resulting treatment dose adjustments, drug switches, and treatment augmentation potentially raise costs further [21–24].

The advent of monoclonal antibody biologics against tumour necrosis factor (anti-TNF), such as infliximab and adalimumab, have improved outcomes in IBD and helped to move treatment goals beyond symptom resolution alone. Nevertheless, widespread uptake has been subject to some reservations about increased risks of infections and bowel surgery, particularly in the elderly [15], and immunogenicity [25]. Non-anti-TNF biologics, such as vedolizumab, ustekinumab, risankizumab, and mirikizumab, have extended treat-to-target options [26, 27] and reduced risks of infection [28] and anti-drug antibodies [29, 30]. However, suboptimal use of such drugs might contribute to a ‘glass ceiling’ effect [31]. Considering different drug mechanisms of action and innovations in treatment strategies could consolidate elusive efforts to break through recognised therapeutic limitations [18, 23, 32, 33].

Decisions on funding of biologic drugs in the UK have considered costs of drugs and cost-effectiveness measured in terms of quality-adjusted life years [34]. This approach, however, may not the capture the impact of active disease on use of health-care resources or rapid disease control and maintenance of remission. Furthermore, anti-TNF biosimilar alternatives have not yet been available long enough to have been included in many health economic assessments, although in Europe biosimilars prescribed for the management of IBD allow savings of 15–75% compared with the original compounds [35]. We used real-world data to examine health-care resource use and to investigate drivers of costs during active disease versus remission. We tested the hypothesis that active disease would be associated with use of more health-care resources and greater costs than remission. We also examined the impacts of age, timing of starting biologics, and the use of non-anti-TNF biologics (ustekinumab and vedolizumab) versus anti-TNFs as first biologic on health-care resources usage and costs.

## Methods

### Study design and population

**Fig. 1:**
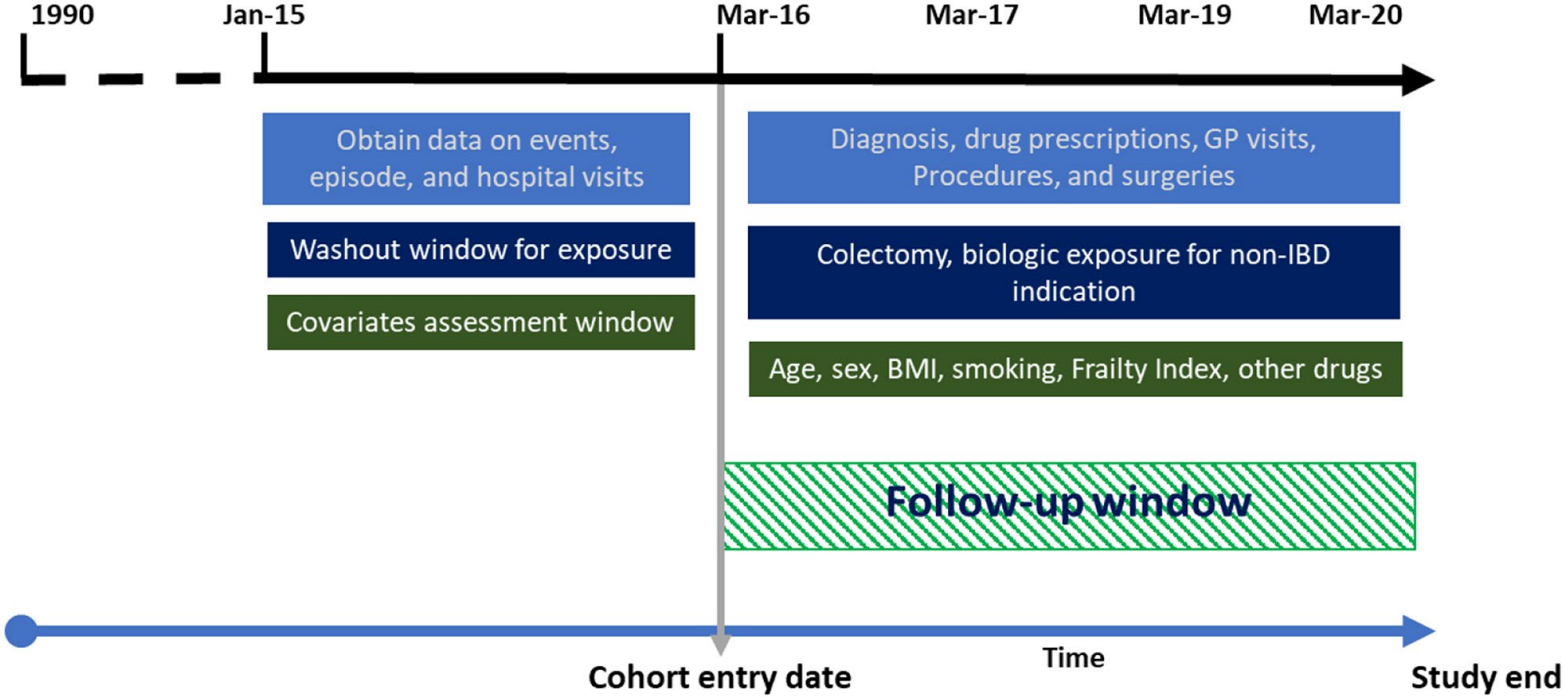
Population identification and selection procedure. Abbreviations: BMI, body-mass index; GP, general (family) practitioner; IBD, inflammatory bowel disease

This was a population-based, retrospective, linked database study of adults (age > 18 years) with IBD in primary and secondary care in northwest London. A pre-study protocol was approved by all investigators for the ethics application. Anonymised data were obtained from the start of 2015 to allow the selection and testing of covariates and ensure patients with a pre-existing IBD diagnosis were excluded. This approach removed uncertainty about previous biologic treatment exposure that would not have been captured for the cohort because the dataset was only linked to the high-cost drug database in 2016. Eligible patients were those who received a diagnosis of incident IBD (a Read code, version 2, in primary care or an ICD 10 code in secondary for UC or CD) between 31 March, 2016, when linkage to the NHS High Cost Drugs Database began, and 31 March, 2020 (Fig. 1). All patients were biologic naïve at inclusion. A small population of patients who had codes for both CD and UC were also included, for whom we adopted the term IBD-undefined to avoid confusion with the term IBD unclassified. The former term aims to capture changes in coding as well as uncertainty of the diagnosis. Data on relevant therapeutic procedures were identified with OPCS Classification of Interventions and Procedure codes (version 4; Appendix Table A1). We excluded patients who had had bowel resections before the recorded use of biologics in 2016, as their treatment might not be representative of the current standard of care.

### Dataset

As part of the integrated care system in northwest London, health and social care data linked by NHS number are collected for around 2.5 million patients registered with an NHS general practice (GP). The data come from more than 400 general practices, mental health service providers, community trusts, and acute providers [36]. Information recorded includes demographics, medical histories, consultation notes, test results, prescriptions, and procedures so that primary, secondary, and other care providers may access a summary of relevant information to aid decision-making. Additional patient-level information on direct commissioners’ costs is also stored.

For this study, we requested access to data via the Discover-NOW Health Data Research Hub for Real World Evidence (Discover) linked dataset, which is hosted by Imperial College Health Partners. All data were deidentified to meet the minimization standards of the Information Standards Boards of NHS Digital, and were stored on a secure server. A governance suppression rule, in which numbers lower than five are annotated as < 5, was applied to further protect anonymity.

### Ethics approval

Ethics approval for the use of the Discover Platform for research was secured from the NHS Health Research Authority in October 2018. The REC reference is 18/WM/0323 and the IRAS project ID is 253,449. The sponsor of the study did not have access to patient-level data.

### Definitions

Disease activity is not recorded in the dataset. Instead, we inferred active disease and remission throughout the study period by review of events: active disease was defined as more than two events within a 12-month period and remission as two or fewer events within a 12-month period. An event was considered to have occurred when one or more of the following activities were recorded: a new prescription of any non-biologic treatment; any dose escalation within the class of drug; addition of another class of drug (any class); a new prescription of any biologic treatment; or a change in biologic treatment (not including change to a biosimilar). A cut off of two events was used to comply with current British Society of Gastroenterology guidelines wherein treatment escalation for disease activity is recommended after two or more steroid courses in 1 year [18]. The first event in each period of active disease was referred to as an index event. Disease status was checked and recorded as changed or unchanged every 12 months.

Drugs were categorised as conventional, anti-TNF biologics, or non-anti-TNF biologics (Appendix Table A2). Initiation of biologics (anti-TNFs and non-anti-TNFs) was separated into early and late (delayed). Early initiation was defined as biologic therapy administered within 3 months of an index event whereas late initiation occurred more than 3 months after an index event or following a second event within a 12-month assessment period.

Terminology for IBD-related resource use is reported as captured in the dataset. Elective inpatient visits refer to planned hospital admissions; non-elective inpatient visits refer to unplanned hospital care after urgent and emergency attendances; outpatient visits refer to clinic attendances and investigations; and primary care visits are attendances at GPs.

### Cost analysis

Discover captures cost data from health-care providers on primary and secondary care. Primary care costs are based on commissioning pricing for different provider services, classified as general care (national-level services), personal care (local-level services), and alternative provider medical services (use of third-party providers), and additional costs like local incentive schemes and out-of-hours services. GP prescribing costs are unavailable at the individual patient level and, therefore, we estimated these from historic data on contract costs, budgets, and sizes and characteristics of practice patient lists. Secondary care costs are based on specific activities (e.g., accident and emergency visits, specific procedures, surgery, length of stay in hospital, etc.) and are available per patient. Community costs are based primarily on block contracts that are split across service lines/activity groupings. Patient-level data are based on the share of a service/activity that an individual uses.

We calculated costs per patient over the study period for direct health-care resource use, overall and separately, for inpatient secondary care (elective hospital admissions, non-elective hospital admissions, and length of stay), outpatient secondary care, primary care, and community care. All costs were calculated in GBP.

### Statistical analysis

The primary outcome was health-care resource use and per-patient costs of care for active disease and remission. Secondary objectives were to compare costs according to disease activity (active disease vs. remission), timing of biologic initiation (early vs. late), and type of biologic received first (non-anti-TNF biologics vs. anti-TNF) in all patients and in two age groups (18–59 years or ≥ 60 years). As this was an exploratory study, descriptive statistics were generated and are presented as frequency distributions for categorical variables and mean values and standard deviations (SDs) for continuous variables. No missing data were imputed. Differences between patients with active disease and those in remission were assessed with the Wilcoxon test. Significance was indicated by p values less than 0.05 and no overlap over 95% confidence intervals. All statistical analyses were performed with R version 2022.02.0.

## Results

### Population demographics

Between 31 March 2016 and 31 March 2020, the number of patients identified with an incident diagnosis of IBD within the study period was 7,733 (Table 1). Of these, 5,872 had UC, 1,427 had CD, and 434 had IBD-undefined. Most patients were younger than 60 years of age and around 44% had comorbidities (Table 1). Roughly half of patients had disease durations of at least 2 years by the end of the study. Active disease was seen mostly in patients in the 18–59-year age group. Throughout the study,  remission was recorded in 19,218 (82%) of 23,488 observations in patients with UC, 4,686 (82%) of 5,708 in patients with CD, and 1,122 (65%) of 1,736 in patients with IBD-undefined. Biologics were prescribed in 392 (5%) patients, two-thirds of whom started them late in the treatment course (Table 1). Within the biologically treated group (*n* = 392), active disease was recorded in 371 (95%) and remission in 21 (5%). The age distribution consisted of 340 (87%) in the younger 18–59-year age group and 55 (14%) in older ≥ 60-year age group.

**Table 1:** Characteristics of patients

Characteristic	UC (*n* = 5,872)	CD (*n* = 1,427)	IBD-undefined (*n* = 434)
**Sex**			
Male	3,023 (51.5%)	691 (48.0%)	229 (53.0%)
Female	2,849 (48.5%)	736 (52.0%)	205 (47.0%)
**Age (years)**			
18‒59 years	3,613 (62%)	1,117 (78.28%)	352 (81.11%)
≥ 60 years	2,259 (39%)	310 (21.72%)	82 (18.89%)
Mean (SD)	52.83 (19.5)	44.03 (18.4)	42.99 (17.6)
Median (range)	55 (18‒103)	39 (18‒100)	39 (18‒88)
**BMI (kg/m** ^**2**^ )*			
Mean (SD)	26.5 (5.8)	25.4 (5.4)	26.0 (5.7)
Median (range)	25.6 (9.2–58.0)	24.8 (10.1–52.5)	25.3 (12.4–45.6)
**Ethnicity (%)**			
Asian	1,524 (26%)	299 (21%)	100 (23%)
Black	344 (6%)	63 (4%)	16 (4%)
Mixed race	131 (2%)	42 (3%)	10 (2%)
White	523 (9%)	144 (10%)	53 (12%)
Other	411 (7%)	105 (7%)	36 (8%)
Not recorded	2,939 (50%)	774 (54%)	219 (50%)
**Smoking status**			
Smoker	1,054 (18%)	314 (22%)	92 (21%)
Ex-smoker	1,015 (17%)	173 (12%)	65 (15%)
Non-smoker	123 (2%)	29 (2%)	8 (2%)
Not recorded	3,680 (63%)	911 (64%)	269 (62%)
**Time from diagnosis to study end (months)**			
< 24	3,000 (51%)	681 (48%)	209 (48%)
≥ 24	2,872 (49%)	746 (52%)	225 (52%)
**Electronic Frailty Index score**			
Mild	808 (14%)	137 (10%)	47 (11%)
Moderate	547 (9%)	70 (5%)	29 (7%)
Severe	405 (7%)	55 (4%)	7 (2%)
Well	3,306 (56%)	911 (64%)	290 (67%)
Not recorded	806 (14%)	254 (18%)	61 (14%)
**At least one comorbidity**			
Number	2,960 (50%)	586 (41%)	177 (40%)
Mean (SD)	2.0 (1.2)	1.8 (1.0)	1.8 (1.2)
Median (range)	2 (1‒7)	1 (1‒8)	1 (1‒7)
**Disease activity status at baseline**			
Active disease	2,040 (35%)	483 (34%)	263 (60%)
18‒59 years	1,409 (69%)	393 (81%)	210 (80%)
≥ 60 years	631 (31%)	90 (19%)	53 (20%)
Remission	3,832 (65%)	944 (66%)	171 (40%)
18‒59 years	2,204 (58%)	724 (77%)	91 (83%)
≥ 60 years	1,628 (42%)	220 (23%)	29 (17%)
**Biologic treatment**			
Yes	177 (3%)	122 (9%)	93 (21%)
Early	70	37	37
Late	107	85	56
No	5,725 (97%)	1,350 (91%)	341 (79%)
**Remission achieved**			
Total number of observations	23,488	5,708	1,736
Observations classified as remission	19,218 (82%)	4,686 (82%)	1,122 (65%)

*Denominators 4,171, 903, and 316 for UC, CD, and IBD-undefined, respectively. Abbreviations: CD, Crohn’s disease; IBD, inflammatory bowel disease; UC, ulcerative colitis

### Health-care resource use

#### Primary analysis

**Fig. 2:**
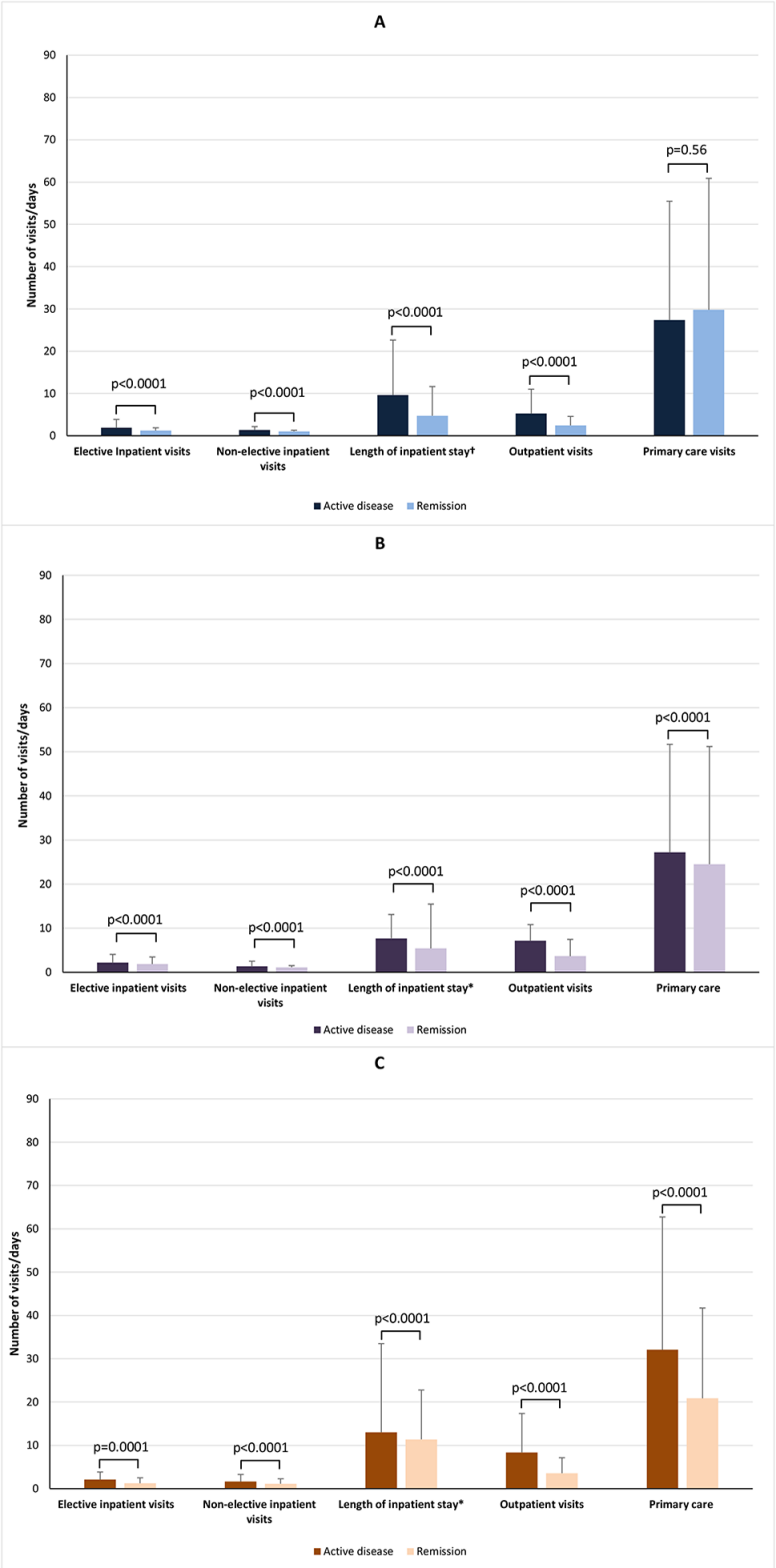
Health-care resource use over the study period by care type and disease activity status. Data are means with standard deviations. (**A**) Ulcerative colitis. (**B**) Crohn’s disease. (**C**) IBD-undefined. *Elective and non-elective hospital visits. Abbreviation: IBD-undefined, diagnosis including codes for both ulcerative colitis and Crohn’s disease

Mean use of all health-care resources and lengths of stay in hospital were significantly greater for patients with active disease than those in remission (all *p* < 0.0001) except for primary care visits in the UC group, which did not differ statistically (Fig. 2). Primary care accounted for most health-care resource use. Other than care for IBD, the most frequent (mean number of visits > 2.00 per patient) reasons for primary care visits among patients with active disease were mental health, measurement of C-reactive protein, and smoking cessation, irrespective of diagnosis; general rheumatology in patients with UC and CD; psoriasis in patients with UC; and depression and anxiety in those with IBD (Appendix Table A3). For patients defined as being in remission, the commonest reasons for primary care visits were mental health for all IBD groups, smoking cessation and depression for UC and CD, and suicide for UC.

#### Subgroup analyses of health-care resource use for active and remission states

##### Age

**Fig. 3:**
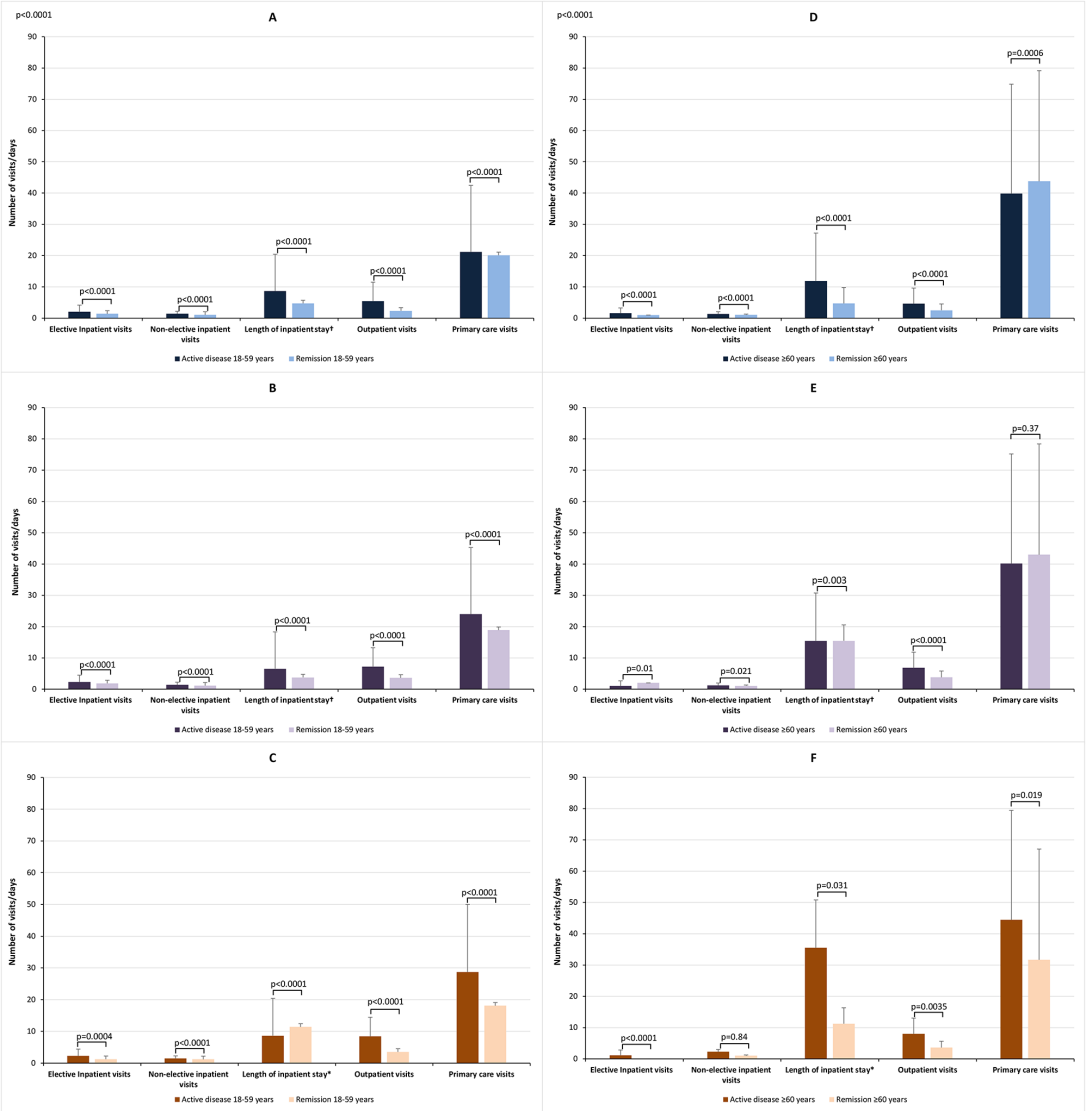
Health-care resource use over the study period by care type, disease activity status, and age group. Data are means with standard deviations. (**A**) Ulcerative colitis, (**B**) Crohn’s disease, and (**C**) IBD-undefined in the 18–59-year age group. (**D**) Ulcerative colitis, (**E**) Crohn’s disease, and (**F**) IBD-undefined in the ≥ 60-year age group. *Elective and non-elective hospital visits. Abbreviation: IBD-undefined: diagnosis including codes for both ulcerative colitis and Crohn’s disease

In the 18–59-year age group, mean frequencies of all types of health-care usage were significantly higher with active disease than with remission (Fig. 3). Hospital stays (elective and non-elective) were significantly longer in patients with active state than remission in the UC and CD groups; the opposite was found in the IBD-undefined group (Fig. 3).

In the 18–59 year age group, the UC group, comparative mean values for active disease versus remission were significantly different: elective inpatient visits 2.02 (SD 2.16) versus 1.40 (SD 0.89), non-elective inpatient visits 1.42 (SD 0.80) versus 1.06 (SD 0.24), length of inpatient stay 8.69 (SD 11.77) days vs. 4.74 (SD 9.27) days, outpatient visits 5.49 (SD 6.02) versus 2.37 (SD 2.22), and primary care visits 21.15 (SD 21.32) versus 20.11 (SD 23.20) (all *p* < 0.0001).

In the CD group, hospital care values for patients age 18–59 years were 2.31 (SD 2.06) versus 1.86 (1.64) for elective inpatient visits, 1.42 (SD 0.95) versus 1.14 (SD 0.39) for non-elective inpatient visits, 6.53 (SD 8.33) days versus 3.76 (SD 5.87) days for length of inpatient stay, 7.23 (SD 6.68) versus 3.61 (SD 3.88) for outpatient visits, and 23.95 (SD 24.73) versus 18.86 (SD 19.67 for primary care visits (all *p* < 0.0001).

The mean values in the IBD-undefined 18–59-year age group were 2.27 (SD 1.87) versus 1.25 (0.50) for elective inpatient visits (*p* = 0.0004), 1.49 (SD 1.10) versus 1.20 (SD 0.42) for non-elective inpatient visits (*p* < 0.0001), 8.67 (SD 10.61) days versus 11.43 (SD 20.00) days for length of stay (*p* < 0.0001), 8.45 (SD 8.66) versus 3.55 (SD 3.15) for outpatient visits (*p* < 0.0001), and 28.70 (SD 27.77) versus 18.10 (SD 18.03) for primary care visits (*p* < 0.0001).

In the ≥ 60-year age group, the UC group, mean per-patient values were significantly higher for the active state than remission for elective inpatient visits (1.61 [SD 1.66] vs. 1.00 [SD 0.00], *p* < 0.0001), non-elective inpatient visits (1.30 [SD 0.76] vs. 1.05 [SD 0.25], *p* < 0.0001), length of inpatient stay (11.92 [SD 15.30] days vs. 4.73 [SD 5.08] days, *p* < 0.0001), and outpatient visits (4.63 [SD 5.00] vs. 2.54 [SD 2.01], *p* < 0.0001), whereas for primary care visits the number was higher with remission (39.82 [SD 35.00] vs. 43.75 [SD 35.40], *p* = 0.0006; Fig. 3).

In the CD group aged ≥ 60 years, mean values were significantly higher with active state than remission for non-elective inpatients visits (1.21 [SD 0.58] vs. 1.08 [SD 0.29], *p* = 0.021) and outpatient visits (6.81 [SD 6.18] vs. 3.79 [SD 3.59], *p* < 0.0001), whereas they were higher with remission for elective inpatient visits (1.00 [SD 0.00] vs. 2.00 [SD 0.00], *p* = 0.01) and duration of inpatient stay (15.44 [SD 15.00] days vs. 15.46 [SD 20.35] days, *p* = 0.003). The mean numbers of primary care visits did not differ significantly (40.18 [SD 39.39] vs. 42.98 [SD 36.61], *p* = 0.37; Fig. 3).

Within the ≥ 60-year IBD-undefined age group, while significantly higher values were seen in active disease for elective inpatient visits (1.17 [SD 0.41] vs. 0 [SD 0.00], *p* < 0.0001), duration of inpatient stay (35.50 [SD 28.25] days vs. 11.25 [SD 6.81] days, *p* = 0.031), outpatient visits (8.03 [SD 10.25] vs. 3.63 [SD 2.08], *p* = 0.0035), and primary care visits (44.44 [SD 37.25] vs. 31.66 [SD 28.04], *p* = 0.0019), the difference for non-elective inpatient visits was not significant (2.27 [SD 3.31] vs. 1.00 [SD 0.00], *p* = 0.84; Fig. 3).

##### Biologic initiation

Comparison of health-care resource use between early and delayed start of biologics showed no significant differences in most of the cohort except for outpatient visit numbers being significantly higher with later biologic use in the IBD-undefined group (mean 6.56 [SD 7.33] vs. 9.15 [SD 7.08], *p* = 0.0049; Appendix Table A4). When we compared the type of biologics used first, the only significant difference in use for any type of health-care resource was for length of hospital stay (elective and non-elective) in the UC group (mean 10.64 [SD 9.30] days vs. 16.65 [SD 18.82], *p* = 0.006; Appendix Table A5).

### Costs

#### Primary analysis

**Fig. 4:**
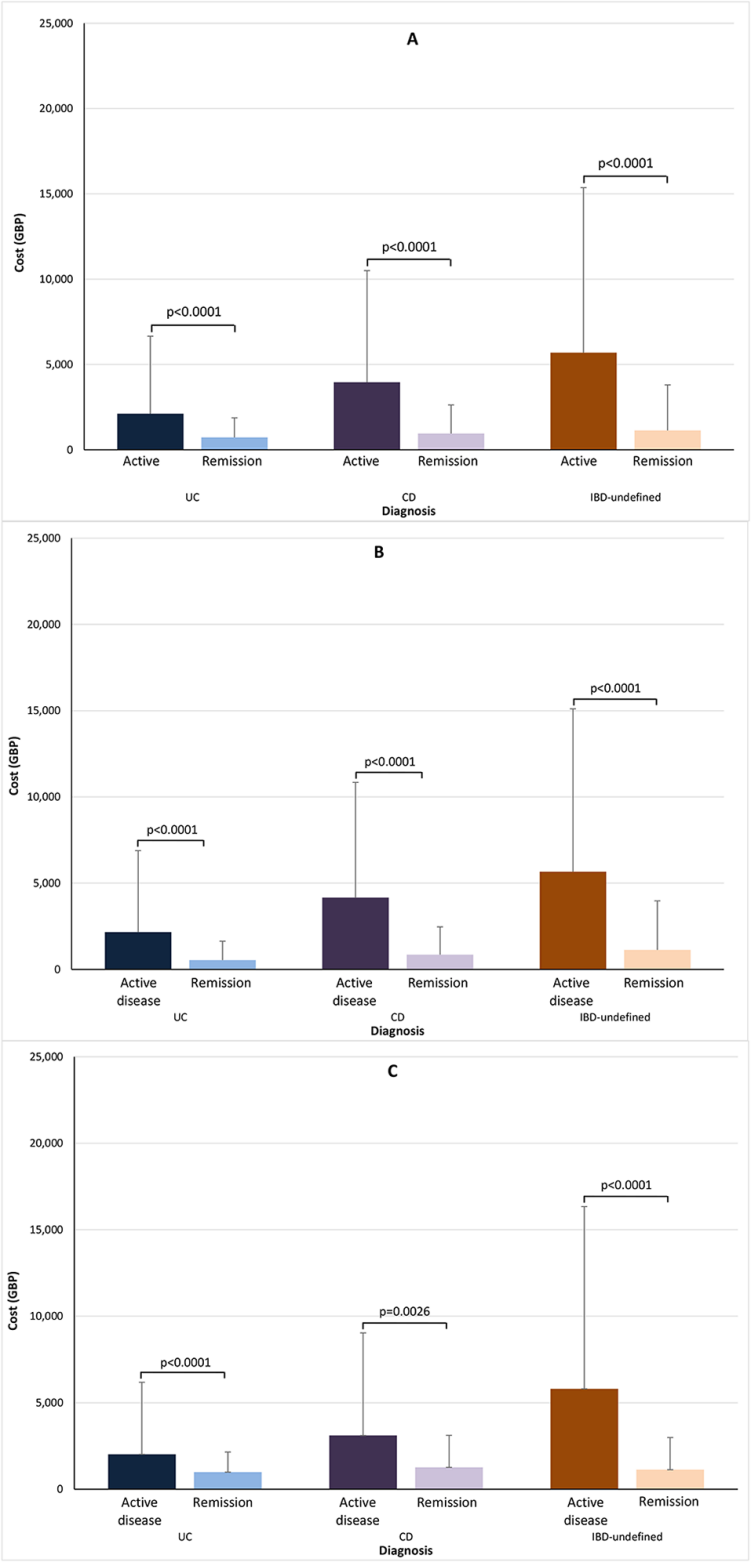
Total per-patient health-care costs over the study period by disease and activity status. Data are means with standard deviations. (**A**) Whole cohort. (**B**) All patients aged 18–59 years. (**C**) All patients aged ≥ 60 years. Abbreviation: IBD-undefined: diagnosis including codes for both ulcerative colitis and Crohn’s disease

**Fig. 5:**
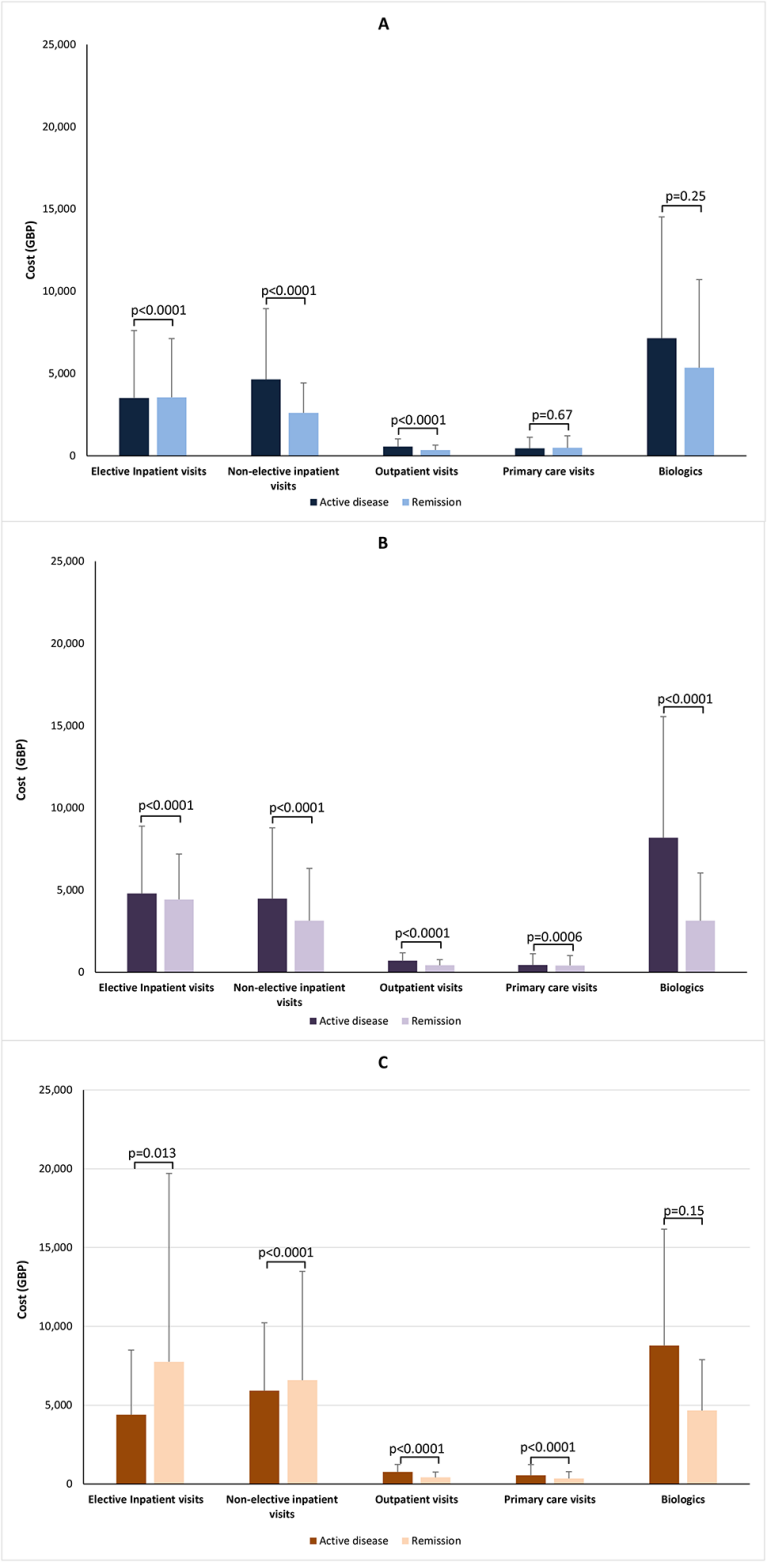
Total per-patient health-care costs over the study period by care type and disease activity status. Data are means with standard deviations. (**A**) Ulcerative colitis. (**B**) Crohn’s disease. (**C**) IBD-undefined. Abbreviation: IBD-undefined: diagnosis including codes for both ulcerative colitis and Crohn’s disease

The mean total per-patient costs of health care were significantly higher among patients with active disease than remission for all ages and by age group (all *p* < 0.0001 except *p* = 0.00026 for CD patents aged ≥ 60 years; Fig. 4). However, when analysing the different care types separately, variation was seen. For UC and CD, significantly higher costs for hospital care (inpatient and outpatient) were associated with the active state while the opposite was true for inpatient care among patients with IBD-undefined (Fig. 5). The mean number of primary care visits were numerically lower with active UC, although not statistically significant (*p* = 0.67), were significantly lower with active CD (*p* = 0.0006), and were significantly higher with active IBD-undefined. Biologics costs were higher with active disease than remission for all diagnoses, but significance was reached for this difference only in the CD group.

#### Subgroup analyses of costs

##### Age

**Fig. 6:**
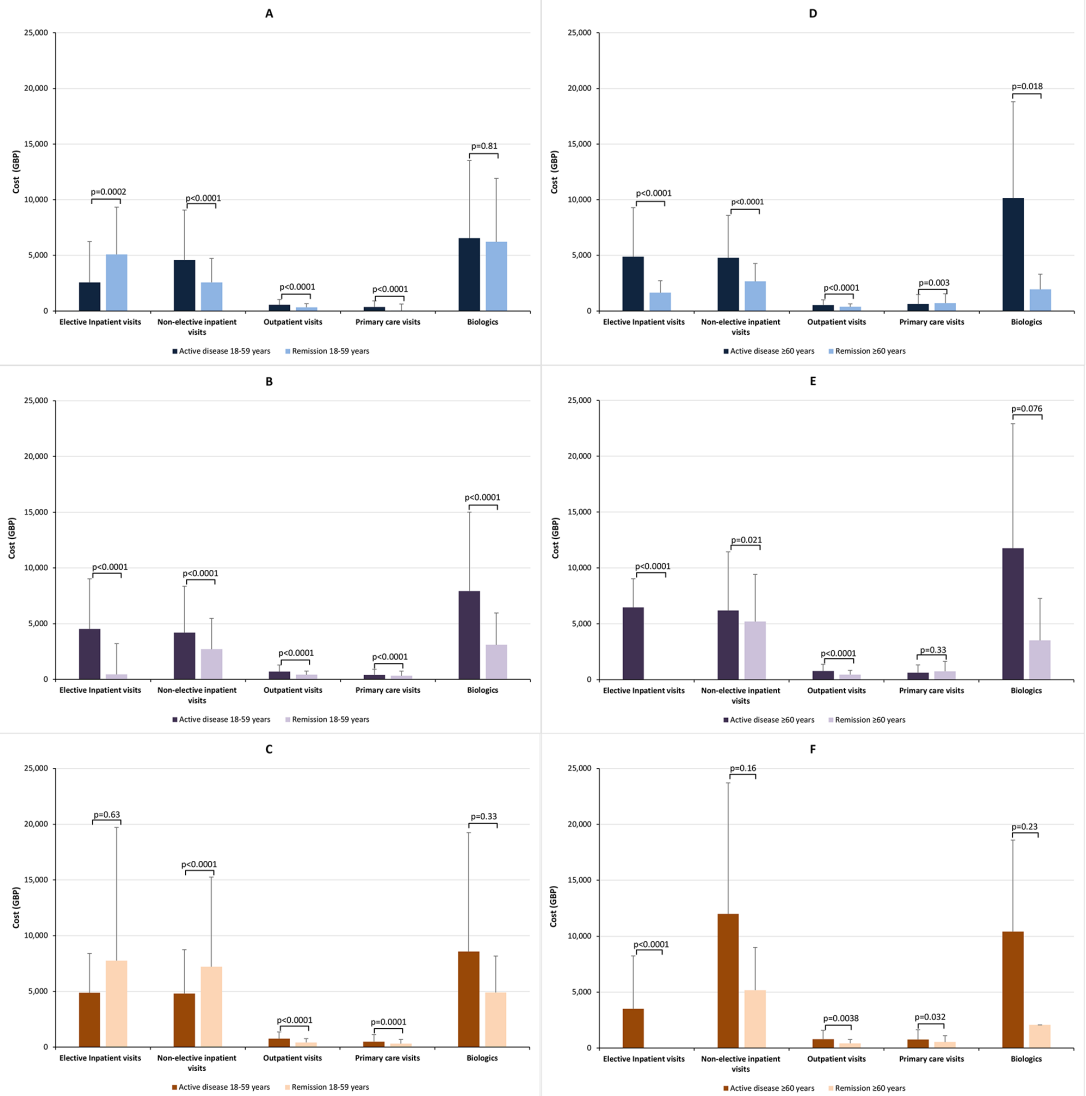
Total per-patient health-care costs over the study period by care type, disease activity status, and age group. Data are means with standard deviations. (**A**) Ulcerative colitis, (**B**) Crohn’s disease, and (**C**) IBD-undefined in the 18–59-year age group. (**D**) Ulcerative colitis, (**E**) Crohn’s disease, and (**F**) IBD-undefined in the ≥ 60-year age group. Abbreviation: IBD-undefined: diagnosis including codes for both ulcerative colitis and Crohn’s disease

For UC, in the 18–59-year age group, the mean per-patient costs were higher with active disease state than remission for non-elective inpatient visits (£4,583 [SD £4,489] vs. £2,582 [SD £2,141]), outpatient visits (£559 [SD £481] vs. £340 [SD £320]), and primary care visits (£357 [SD £558] vs. £32 [SD £599]), whereas the opposite was true for elective inpatient visits (£2,581 [SD £3,660] vs. £5,085 [SD £4,247]) (all, *p* < 0.0001, Fig. 6). The cost of biologics did not differ significantly (mean £6,547 [SD £6,971] vs. £6,213 [SD £5,700], *p* = 0.81). By contrast, the costs for CD were consistently greater with active disease than with remission, and all differences were significant (all *p* < 0.0001, Fig. 6): elective inpatient visits mean £4,581 (SD £4,506) versus £443 (SD £2,759); non-elective inpatient visits £4,183 (SD £4,169) versus £2,702 (SD £2,771); outpatient visits £696 (SD £591) versus £411 (SD £332); primary care visits £401 (SD £509) versus £314 (SD £425); and biologics £7,927 (SD £7,076) versus £3,095 (SD £2,861). For IBD-undefined, costs for elective inpatient visits and biologics differed notably numerically but not statistically (mean £4,863 [SD £3,934] vs. £7,759 [SD £11,948], *p* = 0.63, and £8,567 [SD £9,442] vs. £1,135 [SD £2,842], *p* = 0.33, respectively). Non-elective inpatient visits were significantly lower with active disease than with remission (mean £4,809 [SD £3,954] vs. £7,213 [SD £8,041], *p* < 0.0001) while outpatient and primary care visits were significantly higher with active disease (£761 [SD £599] vs. £418 [£340], *p* < 0.0001, and £493 [SD £627] vs. £310 [SD £374], *p* = 0.0001).

In the ≥ 60-year age group, mean per-patient costs associated with all care types for UC were significantly higher with active disease than with remission (elective inpatient visits mean £4,873 [SD £4,421] vs. £1,646 [SD £1,081], *p* < 0.0001; non-elective inpatient visits £4,479 [SD £3,816] vs. £2,664 [SD £1,608], *p* < 0.0001; outpatient care £547 [SD £463] vs. £375 [SD £270], *p* < 0.0001; and biologics £10,139 [SD £8,667] vs. £1,948 [SD £1,360], *p* = 0.018) except for primary care (£633 [SD £850] vs. £709 [SD £844], *p* = 0.003; Fig. 6). For CD, primary care costs did not differ significantly between disease statuses (mean £632 [SD £691] for active disease vs. £755 [SD £887] for remission, *p* = 0.33) while all other costs were significantly higher with active disease (elective inpatient visits £6,464 [SD £2,575] vs. £0 [SD £0]; non-elective inpatient visits £6,190 [SD £5,254] vs. £5,201 [SD £4,220]; outpatient care £767 [SD £611] vs. £461 [SD £376]; and biologics £11,759 [SD £11,162] vs. £3,517 [SD £3,750]). For IBD-undefined, significantly higher costs were seen with active disease for elective inpatient visits (mean £3,506 [SD £4,730] vs. £0 [SD £0], *p* < 0.0001), outpatient visits (£798 [SD £784] vs. £424 [SD £339], *p* = 0.0038), and primary care visits (£768 [SD £869] vs. £530 [£577], *p* = 0.032). Other costs were numerically higher for patients with active disease but not statistically (non-elective inpatient visits mean £11,989 [SD £11,710] vs. £5,175 [SD £3,825], *p* = 0.16 and biologics £10,409 [SD £8,187] vs. £2,060 [SD £0], *p* = 0.23).

##### Biologic initiation

We found no significant differences in the costs associated with any type of care when assessed by timing of biologic initiation (Appendix Table A6). Costs associated with receiving non-anti-TNFs as the first biologic therapy were significantly more expensive than those of receiving anti-TNFs first (UC mean £11,208 [SD £8,348] vs. £5,068 [SD £5,845], *p* < 0.0001; CD £10,648 [SD £8,370] vs. £6,296 [SD £5,828], *p* = 0.022; IBD-undefined £13,020 [SD £7,022] vs. £6 695 [SD £9,334], *p* < 0.001). However, when direct biologics costs were removed, none of the costs associated with primary or secondary care differed significantly other than that for non-elective inpatient visits among patients with UC and outpatient visits for those with CD (both favoured anti-TNFs; Appendix Table A7).

## Discussion

This large 4-year real-world cohort study shows a substantial burden on the health-care system associated with IBD, wherein more health-care resources are used by patients meeting pre-defined criteria for active disease than by those considered to be in remission. A similar pattern was seen for costs. The greatest health-care resource use is seen for primary care, particularly in patients aged 60 years and older, followed by outpatient care. The overarching driver for costs was inpatient hospital care (elective and non-elective). Total health-care costs were greater in an active state than in remission (all *p* < 0.0001), with the main cost driver being inpatient hospital care visits for active disease. Among patients with UC and IBD-undefined in remission elective inpatient costs were higher than those with active disease.

Understanding the activities driving costs in IBD is important in a two respects: firstly, as the prevalence of the disease continues to rise in high-income countries, anticipating the needs of the population is a prerequisite for planning health services; secondly, as more drugs are marketed for IBD, more-effective therapies and early recognition of non-response facilitates a switch to the next drug to elicit a response, thereby mitigating the likelihood of more costly hospital admissions.

The lower health-care resources cost with remission links economic with clinical arguments for early use of effective therapies to induce and maintain remission in IBD and minimise hospital admissions for active disease, particularly non-elective admissions [17, 37]. In our cohort only 5% of patients (*n* = 392) were prescribed biologics: 144 (37%) received them early, within 3 months of an active disease event, and 248 (63%) received later therapy. We were unable to study the underlying reasons behind prescribing practices due to the retrospective design of the study. However, compared with other cohorts, these figures are low, which suggests undertreatment even beyond the constraints of the definitions of active disease and remission. Other studies within similar time periods showed higher biologic use. In an Israeli study 39% of patients with CD and 15% with UC diagnosed between 2005 and 2020 received biologics [38], while, in a Danish study, among patients diagnosed from 2000 to 2018, 27% with CD and 10% with UC received biologics within 1 year of onset [39]. 

Some studies suggest that costly novel therapies failed to lower the overall cost burden of IBD. Alulis and colleagues [40] reported higher annual costs of treatment among patients who received biologics than for those who did not. Nevertheless, staffing costs were reduced driven by more patients being seen as outpatients. Similarly, Park and colleagues [41] showed that care costs rose with increasing use of biologics after 2014, particularly in elderly IBD patients, where costs were 20–40% higher than those for adults aged 19–54 years. This finding is intriguing, as people older than 60 years are less often treated with biologics [13, 15], similar to the pattern in our cohort. Such findings might be related to co-existing conditions associated with ageing. Burisch and colleagues [42] assessed 5-year direct expenditure profiles for 1,289 patients with IBD across Europe and highlighted that biologics accounted for a significant proportion of direct health-care costs. Concurrently, however, they reported that overall costs decreased over the 5-year period, which they attributed to reduced costs for procedures, hospitalisations, and surgeries despite increased spending on biologicals.

Differing prescribing habits by age group might be driven by safety concerns related to anti-TNF biologics [15], but it seems unlikely that the use of biologics alone, and especially more costly non-anti-TNF biologics, is driving higher treatment costs. Rather, we found that costs for older patients with active disease were high for inpatient hospital care, probably because of the length of stay required. Meanwhile, younger patients with disease in remission accessed the most inpatient care, especially elective hospital visits, which were probably related to attendances for day-care infusions. This is consistent with the reported literature, particularly for UC [43]. More-granular details on the use of care would add value to understand further whether different approaches can reduce the costs associated with more urgent secondary care.

Much of the care burden of patients with IBD in this study fell on primary care, particularly for patients in the 60 years and older age group. While the costs of these visits are low, the numbers of visits require substantial time. In a UK survey of 624 family physicians, 70% reported that they had no formal training in IBD and more than half (52%) expressed low confidence in knowing how to manage disease flares [44]. This could be an important area in which to provide training to GPs and education to patients that could reduce the need for hospital admissions and the number of visits.

Everhov and colleagues [15] showed that increased spending on direct health-care costs can reduce the need for societal support of patients due to sickness absence. Burisch et al. [45] recommended that health-care costs assessments should include indirect costs to better reflect the results of improved disease management, and use registries and big data to assess changes in health-care models in the real world. While we had access to a large real-world dataset, sufficient data on indirect costs were not available to enable such comparisons. We did find that in patients who received biologics, non-drug costs did not increase. We support the improved reporting and use of data on community, social, and other indirect costs.

The Discover dataset is one of Europe’s largest linked longitudinal costed datasets [36], and captures roughly one-third of London’s population, representing wide diversity. It can provide longitudinal health-care data at the patient level based on commissioner pricing for different provider services, linked by patients’ unique NHS identifiers. Primary care data available from birth, including diagnoses, prescriptions, hospital activity, and events such as emergency, outpatient, and social care visits add to the strength of this study. Nevertheless, we caution that there may be some variability in clinical practice between individual centres and other geographical areas that might affect the generalisability of results.

However, this study has some limitations. First, the definitions of active disease and remission were based on indirect assumptions, albeit underpinned by indications for treatment escalation in prevailing guidelines [18]. Shortcomings in the granularity of real-world data is a well-recognised limitation [46]. This issue led to active disease and remission having to be estimated from numbers of events in 12-month periods and remission from the proportion of observations that did not show more than two events in a 12-month period. This approach precluded some analyses, such as accurate calculations of length of remission, which might have been of interest to readers. Additionally, it may have led to an overestimate of the remission rate. Another study using real-world data from UK IBD BioResource calculated that remission at 1 year was 74–76% in patients with CD, dependent on the first-line biologic received, and at 3 years was 50–70% [47]. Norwegian population-based cohort studies suggest clinical remission in 48% of patients with UC and 44% with CD, 5–10 years after diagnosis [2, 3, 48]. Thus, the most appropriate way to record and measure remission should be investigated further. Second, although the Discover dataset includes social care records, methods to calculate societal cost factors at the patient level are still underway. We acknowledge, however, that these social care and wider societal costs are likely to further impact cost-effectiveness in health care. Hansson-Hedblom and colleagues [49] used a cost-effectiveness model to estimate direct and indirect costs of CD in Sweden. They showed that for total costs over a 60-year life-time horizon, indirect costs for loss of productivity accounted for around 50% of disease-associated costs. Another Swedish study showed that mean annual societal costs for adults were increased around threefold with CD and doubled with UC compared with costs for the general population, with 56% and 59%, respectively, being due to loss in productivity [50]. Our aim was to test the hypothesis that active disease is associated with higher use and costs of clinical health care to support clinical practice that delivers timely and effective therapies. We did not assess differences in health-care resource use by subgroups of patients, for example by sex, education, employment, etc., as it was outside the scope of the study. Such population data are stored in the Discover database and following on from our findings could identify subgroups who might benefit most from tailored approaches to treatment. A previous study [51] found multiple sociodemographic and clinical factors within patient subgroups that were associated with high treatment costs, especially unemployment, use of prescribed opiates, and psychiatric and cardiovascular diseases. Of note, data on ethnicity were very poorly recorded in our data set, with records missing for about half of patients. While this level of missing data seems particularly high and the reasons for this are unclear, recording of ethnicity is a known issue across England, particularly in London [52]. 

## Conclusions

In our cohort, only 5% of IBD patients living in northwest London were prescribed biologic therapy. Based on our indirect definition, active disease was associated with significantly greater costs than care of patients in remission. Earlier use of biologics and the use of non-anti-TNF biologics as first-line therapy did not lead to significant increases in overall health-care resource costs, supporting a paradigm of timely and effective therapy. Further analysis of real-world data is warranted to explore subgroups who may benefit most from earlier initiation of biologics, whether uptake of classes of biologics with lower infection risks offer additional benefits for the older age groups, and whether uptake of biologic for this age group may increase with lower non-elective hospital resource use.

## Electronic Supplementary Material

Below is the link to the electronic supplementary material.

Supplementary Material 1

## Data Availability

The data that support the findings of this study are available from Imperial College Health Partners, but restrictions apply to the availability of these data, which were used under license for the current study, and so are not publicly available. Data are however available from the Imperial College Health Partners upon reasonable request and with permission of Professor Naila Arebi.

## Abbreviations

Anti-TNF: Monoclonal antibody against tumour necrosis factor
CD: Crohn’s disease
GP: General practice
IBD: Inflammatory bowel disease
SD: Standard deviation
UC: Ulcerative colitis
