# Supplementary material for: Health-care resource use and costs associated with inflammatory bowel disease in northwest London: a retrospective linked database study
Source: BMC Gastroenterol. 2024 Dec 30;24:480. doi: 10.1186/s12876-024-03559-3 (PMC11684289; doi:10.1186/s12876-024-03559-3)
Supplement: Supplementary file 1 — Supplementary Material 1 [file 12876_2024_3559_MOESM1_ESM.docx]

**SUPPLEMENTARY MATERIAL**

**Health-care resource use and costs associated with inflammatory bowel disease in northwest London: a retrospective linked database study**

Zia UI-Haq, Luiz Causin, Tahereh Kamalati, Durgesh Kahol, Trishan Vaikunthanathan, Charlotte Wong, Naila Arebi

**Table A1: Relevant OPCS Classification of Interventions and Procedure codes**

| **Code** | **Description** | **Group** |
| --- | --- | --- |
| G310 | Conversion from previous anastomosis of stomach to duodenum | Intestinal Bypass |
| G311 | Bypass of stomach by anastomosis of oesophagus to duodenum | Intestinal Bypass |
| G312 | Bypass of stomach by anastomosis of stomach to duodenum | Intestinal Bypass |
| G313 | Revision of anastomosis of stomach to duodenum | Intestinal Bypass |
| G314 | Conversion to anastomosis of stomach to duodenum | Intestinal Bypass |
| G315 | Closure of connection of stomach to duodenum | Intestinal Bypass |
| G316 | Attention to connection of stomach to duodenum | Intestinal Bypass |
| G318 | Other specified connection of stomach to duodenum | Intestinal Bypass |
| G319 | Unspecified connection of stomach to duodenum | Intestinal Bypass |
| G320 | Conversion from previous anastomosis of stomach to transposed jejunum | Intestinal Bypass |
| G321 | Bypass of stomach by anastomosis of stomach to transposed jejunum | Intestinal Bypass |
| G322 | Revision of anastomosis of stomach to transposed jejunum | Intestinal Bypass |
| G323 | Conversion to anastomosis of stomach to transposed jejunum | Intestinal Bypass |
| G324 | Closure of connection of stomach to transposed jejunum | Intestinal Bypass |
| G325 | Attention to connection of stomach to transposed jejunum | Intestinal Bypass |
| G328 | Other specified connection of stomach to transposed jejunum | Intestinal Bypass |
| G329 | Unspecified connection of stomach to transposed jejunum | Intestinal Bypass |
| G330 | Conversion from previous anastomosis of stomach to jejunum NEC | Intestinal Bypass |
| G331 | Bypass of stomach by anastomosis of stomach to jejunum NEC | Intestinal Bypass |
| G332 | Revision of anastomosis of stomach to jejunum NEC | Intestinal Bypass |
| G333 | Conversion to anastomosis of stomach to jejunum NEC | Intestinal Bypass |
| G334 | Open reduction of intussusception of gastroenterostomy | Intestinal Bypass |
| G335 | Closure of connection of stomach to jejunum NEC | Intestinal Bypass |
| G336 | Attention to connection of stomach to jejunum | Intestinal Bypass |
| G338 | Other specified other connection of stomach to jejunum | Intestinal Bypass |
| G339 | Unspecified other connection of stomach to jejunum | Intestinal Bypass |
| G491 | Gastroduodenectomy | Small bowel resection |
| G492 | Total excision of duodenum | Small bowel resection |
| G493 | Partial excision of duodenum | Small bowel resection |
| G498 | Other specified excision of duodenum | Small bowel resection |
| G499 | Unspecified excision of duodenum | Small bowel resection |
| G511 | Bypass of duodenum by anastomosis of stomach to jejunum | Intestinal Bypass |
| G512 | Bypass of duodenum by anastomosis of duodenum to duodenum | Intestinal Bypass |
| G513 | Bypass of duodenum by anastomosis of duodenum to jejunum | Intestinal Bypass |
| G514 | Bypass of duodenum by anastomosis of duodenum to colon | Intestinal Bypass |
| G518 | Other specified bypass of duodenum | Intestinal Bypass |
| G519 | Unspecified bypass of duodenum | Intestinal Bypass |
| G531 | Open biopsy of lesion of duodenum | Intestinal Bypass |
| G532 | Closure of perforation of duodenum NEC | Repair of intestinal perforation |
| G533 | Open removal of foreign body from duodenum | Repair of intestinal perforation |
| G534 | Open insertion of tubal prosthesis into duodenum | Repair of intestinal perforation |
| G535 | Incision of duodenum NEC | Repair of intestinal perforation |
| G536 | Correction of malrotation of duodenum | Repair of intestinal perforation |
| G538 | Other specified other open operations on duodenum | Repair of intestinal perforation |
| G539 | Unspecified other open operations on duodenum | Repair of intestinal perforation |
| G581 | Total jejunectomy and anastomosis of stomach to ileum | Excision of jejunum |
| G582 | Total jejunectomy and anastomosis of duodenum to ileum | Excision of jejunum |
| G583 | Total jejunectomy and anastomosis of duodenum to colon | Excision of jejunum |
| G584 | Partial jejunectomy and anastomosis of jejunum to ileum | Excision of jejunum |
| G585 | Partial jejunectomy and anastomosis of duodenum to colon | Excision of jejunum |
| G588 | Other specified excision of jejunum | Excision of jejunum |
| G589 | Unspecified excision of jejunum | Excision of jejunum |
| G601 | Creation of jejunostomy | Stoma formation |
| G602 | Refashioning of jejunostomy | stoma revision |
| G603 | Closure of jejunostomy | stoma revision |
| G608 | Other specified artificial opening into jejunum | stoma revision |
| G609 | Unspecified artificial opening into jejunum | stoma revision |
| G633 | Closure of perforation of jejunum | stoma revision |
| G691 | Ileectomy and anastomosis of stomach to ileum | Excision of ileum |
| G692 | Ileectomy and anastomosis of duodenum to ileum | Excision of ileum |
| G693 | Ileectomy and anastomosis of ileum to ileum | Excision of ileum |
| G694 | Ileectomy and anastomosis of ileum to colon | Excision of ileum |
| G698 | Other specified excision of ileum | Excision of ileum |
| G699 | Unspecified excision of ileum | Excision of ileum |
| G711 | Bypass of ileum by anastomosis of jejunum to ileum | Intestinal Bypass |
| G712 | Bypass of ileum by anastomosis of ileum to ileum | Intestinal Bypass |
| G713 | Bypass of ileum by anastomosis of ileum to caecum | Intestinal Bypass |
| G714 | Bypass of ileum by anastomosis of ileum to transverse colon | Intestinal Bypass |
| G715 | Bypass of ileum by anastomosis of ileum to colon NEC | Intestinal Bypass |
| G721 | Anastomosis of ileum to caecum | Intestinal Bypass |
| G722 | Anastomosis of ileum to transverse colon | Intestinal Bypass |
| G723 | Anastomosis of ileum to colon NEC | Intestinal Bypass |
| G724 | Anastomosis of ileum to rectum | Intestinal Bypass |
| G725 | Anastomosis of ileum to anus and creation of pouch HFQ | Intestinal Bypass |
| G728 | Other specified other connection of ileum | Intestinal Bypass |
| G729 | Unspecified other connection of ileum | Intestinal Bypass |
| G731 | Revision of anastomosis of ileum | Intestinal Bypass |
| G731 | Revision of anastomosis of ileum | Other |
| G732 | Closure of anastomosis of ileum | Other |
| G733 | Resection of ileostomy | Other |
| G734 | Resection of ileocolic anastomosis | Other |
| G738 | Other specified attention to connection of ileum | Other |
| G739 | Unspecified attention to connection of ileum | Other |
| G741 | Creation of continent ileostomy | Stoma formation |
| G742 | Creation of temporary ileostomy | Stoma formation |
| G743 | Creation of defunctioning ileostomy | Stoma formation |
| G748 | Other specified creation of artificial opening into ileum | Stoma formation |
| G749 | Unspecified creation of artificial opening into ileum | Stoma formation |
| G751 | Refashioning of ileostomy | stoma revision |
| G752 | Repair of prolapse of ileostomy | stoma revision |
| G753 | Closure of ileostomy | stoma revision |
| G754 | Dilation of ileostomy | stoma revision |
| G755 | Reduction of prolapse of ileostomy | stoma revision |
| G756 | Resiting of ileostomy | stoma revision |
| G758 | Other specified attention to artificial opening into ileum | stoma revision |
| G759 | Unspecified attention to artificial opening into ileum | stoma revision |
| G782 | Strictureplasty of ileum | stoma revision |
| G784 | Closure of perforation of ileum | Repair of intestinal perforation |
| H041 | Panproctocolectomy and ileostomy | Colorectal resection (other) |
| H042 | Panproctocolectomy and anastomosis of ileum to anus and creation of pouch HFQ | Colorectal resection (other) |
| H043 | Panproctocolectomy and anastomosis of ileum to anus NEC | Colorectal resection (other) |
| H048 | Other specified total excision of colon and rectum | Colorectal resection (other) |
| H049 | Unspecified total excision of colon and rectum | Colorectal resection (other) |
| H051 | Total colectomy and anastomosis of ileum to rectum | Colorectal resection (other) |
| H052 | Total colectomy and ileostomy and creation of rectal fistula HFQ | Colorectal resection (other) |
| H053 | Total colectomy and ileostomy NEC | Colorectal resection (other) |
| H058 | Other specified total excision of colon | Colorectal resection (other) |
| H059 | Unspecified total excision of colon | Colorectal resection (other) |
| H061 | Extended right hemicolectomy and end to end anastomosis | Colectomy: right |
| H062 | Extended right hemicolectomy and anastomosis of ileum to colon | Colectomy: right |
| H063 | Extended right hemicolectomy and anastomosis NEC | Colectomy: right |
| H064 | Extended right hemicolectomy and ileostomy HFQ | Colectomy: right |
| H065 | Extended right hemicolectomy and end to side anastomosis | Colectomy: right |
| H068 | Other specified extended excision of right hemicolon | Colectomy: right |
| H069 | Unspecified extended excision of right hemicolon | Colectomy: right |
| H071 | Right hemicolectomy and end to end anastomosis of ileum to colon | Colectomy: right |
| H072 | Right hemicolectomy and side to side anastomosis of ileum to transverse colon | Colectomy: right |
| H073 | Right hemicolectomy and anastomosis NEC | Colectomy: right |
| H074 | Right hemicolectomy and ileostomy HFQ | Colectomy: right |
| H075 | Right hemicolectomy and end to side anastomosis | Colectomy: right |
| H078 | Other specified other excision of right hemicolon | Colectomy: right |
| H079 | Unspecified other excision of right hemicolon | Colectomy: right |
| H081 | Transverse colectomy and end to end anastomosis | Colectomy: right |
| H082 | Transverse colectomy and anastomosis of ileum to colon | Colectomy: right |
| H083 | Transverse colectomy and anastomosis NEC | Colectomy: right |
| H084 | Transverse colectomy and ileostomy HFQ | Colectomy: right |
| H085 | Transverse colectomy and exteriorisation of bowel NEC | Colectomy: right |
| H086 | Transverse colectomy and end to side anastomosis | Colectomy: right |
| H088 | Other specified excision of transverse colon | Colectomy: right |
| H089 | Unspecified excision of transverse colon | Colectomy: right |
| H091 | Left hemicolectomy and end to end anastomosis of colon to rectum | Colectomy: left (including anterior resection) |
| H092 | Left hemicolectomy and end to end anastomosis of colon to colon | Colectomy: left (including anterior resection) |
| H093 | Left hemicolectomy and anastomosis NEC | Colectomy: left (including anterior resection) |
| H094 | Left hemicolectomy and ileostomy HFQ | Colectomy: left (including anterior resection) |
| H095 | Left hemicolectomy and exteriorisation of bowel NEC | Colectomy: left (including anterior resection) |
| H096 | Left hemicolectomy and end to side anastomosis | Colectomy: left (including anterior resection) |
| H098 | Other specified excision of left hemicolon | Colectomy: left (including anterior resection) |
| H099 | Unspecified excision of left hemicolon | Colectomy: left (including anterior resection) |
| H101 | Sigmoid colectomy and end to end anastomosis of ileum to rectum | Colectomy: left (including anterior resection) |
| H102 | Sigmoid colectomy and anastomosis of colon to rectum | Colectomy: left (including anterior resection) |
| H103 | Sigmoid colectomy and anastomosis NEC | Colectomy: left (including anterior resection) |
| H104 | Sigmoid colectomy and ileostomy HFQ | Colectomy: left (including anterior resection) |
| H105 | Sigmoid colectomy and exteriorisation of bowel NEC | Colectomy: left (including anterior resection) |
| H106 | Sigmoid colectomy and end to side anastomosis | Colectomy: left (including anterior resection) |
| H108 | Other specified excision of sigmoid colon | Colectomy: left (including anterior resection) |
| H109 | Unspecified excision of sigmoid colon | Colectomy: left (including anterior resection) |
| H111 | Colectomy and end to end anastomosis of colon to colon NEC | Colectomy: subtotal |
| H112 | Colectomy and side to side anastomosis of ileum to colon NEC | Colectomy: subtotal |
| H113 | Colectomy and anastomosis NEC | Colectomy: subtotal |
| H114 | Colectomy and ileostomy NEC | Colectomy: subtotal |
| H115 | Colectomy and exteriorisation of bowel NEC | Colectomy: subtotal |
| H116 | Colectomy and end to side anastomosis NEC | Colectomy: subtotal |
| H118 | Other specified other excision of colon | Colectomy: subtotal |
| H119 | Unspecified other excision of colon | Colectomy: subtotal |
| H131 | Bypass of colon by anastomosis of ileum to colon | Colectomy: subtotal |
| H132 | Bypass of colon by anastomosis of caecum to sigmoid colon | Colectomy: subtotal |
| H133 | Bypass of colon by anastomosis of transverse colon to sigmoid colon | Colectomy: subtotal |
| H134 | Bypass of colon by anastomosis of transverse colon to rectum | Colectomy: subtotal |
| H135 | Bypass of colon by anastomosis of colon to rectum NEC | Colectomy: subtotal |
| H138 | Other specified bypass of colon | Colectomy: subtotal |
| H139 | Unspecified bypass of colon | Colectomy: subtotal |
| H151 | Loop colostomy | stoma formation |
| H152 | End colostomy | stoma formation |
| H153 | Refashioning of colostomy | stoma revision |
| H154 | Closure of colostomy | stoma revision |
| H155 | Dilation of colostomy | stoma revision |
| H156 | Reduction of prolapse of colostomy | stoma revision |
| H158 | Other specified other exteriorisation of colon | stoma revision |
| H159 | Unspecified other exteriorisation of colon | stoma revision |
| H331 | Abdominoperineal excision of rectum and end colostomy | Colectomy: left (including anterior resection) |
| H332 | Proctectomy and anastomosis of colon to anus | Colectomy: left (including anterior resection) |
| H333 | Anterior resection of rectum and anastomosis of colon to rectum using staples | Colectomy: left (including anterior resection) |
| H334 | Anterior resection of rectum and anastomosis NEC | Colectomy: left (including anterior resection) |
| H335 | Rectosigmoidectomy and closure of rectal stump and exteriorisation of bowel | Hartmann’s procedure |
| H336 | Anterior resection of rectum and exteriorisation of bowel | Colectomy: left (including anterior resection) |
| H337 | Perineal resection of rectum HFQ | Colectomy: left (including anterior resection) |
| H338 | Other specified excision of rectum | Colectomy: left (including anterior resection) |
| H339 | Unspecified excision of rectum | Colectomy: left (including anterior resection) |
| H481 | Excision of polyp of anus | Anal excision Grp |
| H482 | Excision of skin tag of anus | Anal excision Grp |
| H483 | Excision of perianal wart | Anal excision Grp |
| H488 | Other specified excision of lesion of anus | Anal excision Grp |
| H489 | Unspecified excision of lesion of anus | Anal excision Grp |
| H551 | Laying open of low anal fistula | Perianal interventions |
| H552 | Laying open of high anal fistula | Perianal interventions |
| H553 | Laying open of anal fistula NEC | Perianal interventions |
| H554 | Insertion of seton into high anal fistula and partial laying open of track HFQ | Perianal interventions |
| H555 | Fistulography of anal fistula | Perianal interventions |
| H556 | Probing of perineal fistula | Perianal interventions |
| H557 | Repair of anal fistula using plug | Perianal interventions |
| H558 | Other specified other operations on perianal region | Perianal interventions |
| H559 | Unspecified other operations on perianal region | Perianal interventions |
| H581 | Drainage of ischiorectal abscess | abscess related |
| H582 | Drainage of perianal abscess | abscess related |
| H583 | Drainage of perirectal abscess | abscess related |
| H588 | Other specified drainage through perineal region | abscess related |
| H589 | Unspecified drainage through perineal region | abscess related |
| H628 | Other specified other operations on bowel | abscess related |
| H629 | Unspecified other operations on bowel | abscess related |
| H661 | Excision of ileoanal pouch | abscess related |
| H662 | Revision of ileoanal pouch | abscess related |
| H668 | Other specified therapeutic operations on ileoanal pouch | abscess related |
| H669 | Unspecified therapeutic operations on ileoanal pouch | abscess related |

**Table A2: Categorisations of drugs**

| **Conventional treatments** | **Anti-TNF biologics** | **Newer biologics** |
| --- | --- | --- |
| **Mesalazine (5-aminosalicylic acid)** | **Biologics** | **Biologics** |
| Octasa | **Infliximab** (Remicade) | Ustekinumab (Stelara) |
| Pentasa | Remsima (biosimilar) | Vedolizumab (Entyvio) |
| Sulfasalazine | Inflectra (biosimilar) | **Small-molecule Inhibitor** |
| Asacol | **Adalimumab** (Humira) | Tofacitinib (Xeljanz) |
| Mezavant | Amgevita (biosimilar) |  |
| Salofalk | Solymbic (biosimilar) |  |
| **Steroids** | Imraldi (biosimilar) |  |
| Prednisolone | Cyltezo (biosimilar) |  |
| Budesonide | Halimatoz (biosimilar) |  |
| Beclomethasone | Hefiya (biosimilar) |  |
| **Immunomodulators** | Hyrimoz (biosimilar) |  |
| Azathioprine | Hulio (biosimilar) |  |
| Mercaptopurine | Idacio (biosimilar) |  |
| Methotrexate | Kromeya (biosimilar) |  |
| Tacrolimus | **Golimumab** (Simponi) |  |
| **Antibiotics** |  |  |
| Ciprofloxacin |  |  |
| Metronidazole |  |  |

Table A3: Most frequent causes of primary care visits and surgery by diagnosis

|  | UC |  | CD |  | IBD-unclassified |  |
| --- | --- | --- | --- | --- | --- | --- |
|  | Active disease | Remission | Active disease | Remission | Active disease | Remission |
| **Primary care** | | | | | | |
| C-reactive protein | 2.22 (2.44) | 1.75 (1.35) | 2.58 (2.82) | 1.89 (2.11) | 2.49 (2.28) | 1.92 (1.32) |
| Smoking | 2.37 (2.44) | 2.62 (2.13) | 2.33 (1.98) | 2.30 (1.67) | 2.54 (2.54) | 1.93 (1.23) |
| Faecal calprotectin | 1.38 (0.73) | 1.35 (0.72) | 1.56 (0.96) | 1.44 (0.89) | 1.64 (1.13) | 1.31 (0.69) |
| General rheumatology | 2.55 (2.67) | 1.90 (1.67) | 2.75 (3.26) | 1.68 (1.63) | 1.82 (1.33) | 1.95 (1.58) |
| Mental health | 3.05 (4.72) | 3.16 (4.44) | 2.38 (1.42) | 2.17 (2.19) | 2.35 (2.73) | 2.69 (2.10) |
| Depression | 1.62 (1.20) | 2.11 (2.24) | 1.71 (1.35) | 2.55 (3.08) | 2.28 (1.78) | 1.65 (1.11) |
| Anxiety | 1.73 (1.70) | 1.81 (2.07) | 1.50 (1.00) | 1.83 (1.71) | 2.07 (2.01) | 1.66 (1.03) |
| Bowel general | 1.33 (0.53) | 1.41 (0.73) | 2.00 (1.41) | 1.70 (1.10) | 1.00 (0.00) | 1.25 (0.50) |
| Psoriasis | 2.96 (4.00) | 1.92 (1.70) | 1.45 (0.69) | 1.45 (0.69) | 1.66 (0.58) | 1.00 (0.00) |
| Suicide | 1.72 (1.39) | 2.34 (2.95) | 1.87 (0.99) | 1.30 (0.67) | 1.50 (0.58) | 1.50 (1.00) |
| Abdominal scan | 1.00 (0.00) | 1.04 (0.20) | 1.09 (0.30) | 1.00 (0.00) | 1.00 (0.00) | 1.33 (0.58) |
| Weight/BMI | 1.07 (0.28) | 1.22 (0.70) | 1.00 (0.00) | 1.00 (0.00) | 1.00 (0.00) | 0.00 (0.00) |
| **Surgery** | | | | | | |
| Colectomy: right | 1.00 (0) | 0 (0) | 1.00 (0) | 1.00 (0) | 1.00 (0) | 0 (0) |
| Stoma formation | 0 (0) | 0 (0) | 1.00 (0) | 1.00 (0) | 1.00 (0) | 1.00 (0) |
| stoma revision | 1.00 (0) | 0 (0) | 1.00 (0.38) | 1.00 (0) | 1.33 (0.58) | 1.00 (0) |
| Colorectal resection (other) | 1.12 (0.33) | 1.00 (0) | 1.00 (0) | 0 (0) | 1.00 (0) | 1.00 (0) |
| Excision of ileum | 0 (0) | 0 (0) | 1.00 (0) | 1.00 (0) | 0 (0) | 1.00 (0) |
| Perianal interventions | 0 (0) | 0 (0) | 1.00 (0) | 1.50 (0.71) | 2.00 (0) | 1.00 (0) |
| Colectomy: subtotal | 1.00 (0) | 0 (0) | 1.00 (0) | 0 (0) | 0 (0) | 0 (0) |
| Colectomy: left (including anterior resection) | 0 (0) | 0 (0) | 0 (0) | 0 (0) | 1.00 (0) | 1.00 (0) |
| Anal excision Grp | 1.00 (0) | 0 (0) | 0 (0) | 0 (0) | 0 (0) | 0 (0) |

Data are mean (SD). Abbreviations: CD, Crohn’s disease; IBD-undefined, patients with codes for both CD and UC; UC, ulcerative colitis.

Table A4: Comparison of health-care resource use between early and late start of biologics

| **Type of care** | **UC** | | | **CD** | | | **IBD-undefined** | | |
| --- | --- | --- | --- | --- | --- | --- | --- | --- | --- |
|  | Early biologic start (n=70) | Late biologic start (n=107) | P values | Early biologic start (n=37) | Late biologic start (n=85) | P values | Early biologic start (n=37) | Late biologic start (n=56) | P values |
| Elective inpatient visits | 2.00 (1.15) | 2.39 (2.06) | 0.77 | 2.79 (2.50) | 2.48 (1.65) | 0.92 | 2.45 (1.69) | 2.19 (1.38) | 0.86 |
| Non-elective inpatient visits | 1.68 (0.78) | 1.70 (0.82) | 0.28 | 1.36 (0.633) | 1.43 (0.84) | 0.081 | 2.56 (2.85) | 1.12 (0.33) | 0.16 |
| Length of inpatient stay (days)* | 17.51 (18.40) | 12.14 (12.50) | 0.78 | 6.63 (7.75) | 4.52 (10.45) | 0.24 | 13.65 (17.24) | 5.72 (5.50) | 0.078 |
| Outpatient visits | 6.43 (6.95) | 6.79 (5.62) | 0.49 | 6.61 (5.08) | 7.52 (6.44) | 0.28 | 6.56 (7.33) | 9.15 (7.08) | **0.0049** |
| Primary care visits | 19.53 (14.89) | 21.28 (20.35) | 0.83 | 17.25 (17.03) | 18.12 (15.05) | 0.69 | 21.03 (24.25) | 20.57 (20.46) | 0.65 |

Data are mean (SD). *Elective and non-elective Abbreviations: CD, Crohn’s disease; IBD-undefined, patients with codes for both CD and UC; UC, ulcerative colitis.

Table A5: Comparison of health-care resource use between patients who started biologic therapy with newer biologics or anti-TNFs

| **Care type** | **UC** | | | **CD** | | | **IBD-undefined** | | |
| --- | --- | --- | --- | --- | --- | --- | --- | --- | --- |
|  | Newer Advance Therapy first line (n=28) | Anti-TNF first line (n=145) | P values | Newer Advance Therapy first line (n=14) | Anti-TNF first line (n=147) | P values | Newer Advance Therapy first line (n=8) | Anti-TNF first line (n=99) | P values |
| Elective inpatient visits | 4.57 (4.12) | 2.00 (1.41) | 0.34 | 1.33 (0.58) | 3.12 (2.24) | 0.59 | 1.00 (0.00) | 2.86 (2.03) | 0.46 |
| Non-elective inpatient visits | 1.91 (1.22) | 1.85 (1.17) | 0.35 | 1.00 (0.00) | 1.55 (1.06) | 0.37 | 1.5 (0.71) | 2.08 (2.37) | 0.57 |
| Length of inpatient stay (days)* | 10.64 (9.30) | 16.65 (18.82) | **0.006** | 5.14 (3.29) | 5.94 (9.77) | 0.27 | 5.75 (4.50) | 11.88 (14.58) | 0.64 |
| Outpatient visits | 10.96 (10.26) | 10.68 (9.41) | 0.45 | 13.78 (6.42) | 10.72 (9.13) | 0.18 | 11.38 (10.64) | 13.85 (12.62) | 0.59 |
| Primary care visits | 37.94 (28.87) | 41.56 (40.54) | 0.66 | 28.31 (17.46) | 31.60 (35.04) | 0.22 | 44.25 (25.08) | 44.89 (40.24) | 0.33 |

Data are mean SD. *Elective and non-elective. Abbreviations: anti-TNF, monoclonal antibody tumour necrosis factor alpha inhibitor. CD, Crohn’s disease; IBD-undefined, patients with codes for both Crohn’s disease and ulcerative colitis; UC, ulcerative colitis.

Table A6: Per-patient cost of health-care usage by diagnosis, timing of biologic treatment, and health-care activity

| **Type of care** | **UC** | | | | **CD** | | | **IBD-undefined** | | |  |
| --- | --- | --- | --- | --- | --- | --- | --- | --- | --- | --- | --- |
|  | Early start (n=70) | Late start (n=107) | P values | Early start (n=37) | | Late start (n=85) | P values | Early start (n=37) | Late start (n=56) | P values | |
| Elective inpatient visits | 2,138 (4582) | 1,143 (2,552) | 0.78 | 1,655 (2950) | | 518 (1,614) | 0.23 | 1,245 (3462) | 2,017 (3,674) | 0.39 | |
| Non-elective inpatient visits | 6,615 (5,167) | 6,318 (54,15) | 0.96 | 4,975 (3,668) | | 3,921 (3,616) | 0.14 | 8,751 (9,433) | 3,568 (2,459) | 0.55 | |
| Outpatient visits | 482 (432) | 553 (486) | 0.33 | 513 (439) | | 675 (620) | 0.65 | 638 (680) | 647 (513) | 0.17 | |
| Primary care visits | 378 (421) | 365 (392) | 0.75 | 338 (378) | | 295 (292) | 0.59 | 305 (398) | 353 (521) | 0.71 | |
| Mean of all mean visit costs | 5,187 (5,658) | 4,655 (5,408) | 0.45 | 3,990 (4,505) | | 3,274 (3,246) | 0.56 | 6,185 (8,294) | 3,843 (3,371) | 0.67 | |
| Biologic treatment | 6,110 (6,427) | 4,917 (4,999) | 0.13 | 5,500 (4,555) | | 5,179 (5,541) | 0.28 | 6,267 (6,534) | 5,441 (7,249) | 0.24 | |

Data are number (SD; range). Abbreviations: CD, Crohn’s disease; IBD-undefined, patients with codes for both Crohn’s disease and ulcerative colitis; UC, ulcerative colitis.

Table A7: Per-patient costs of health-care usage by diagnosis, timing of biologic treatment, and type of first-line biologic in all patients and by age group

| **Health-care type** | **UC** | | | **CD** | | | **IBD-undefined** | | |
| --- | --- | --- | --- | --- | --- | --- | --- | --- | --- |
|  | Newer biologics (n=28) | Anti-TNFs (n=145) | P values | Newer biologics (n=14) | Anti-TNFs (n=147) | P values | Newer biologics (n=8) | Anti-TNFs (n=99) | P values |
| Elective inpatient visits | 7,654 (10,132) | 2,894 (3,480) | 0.72 | 3,966 (1,390) | 3,623 (3,212) | 0.49 | 3,901 (4,887) | 7,720 (6,784) | 0.19 |
| Non-elective inpatient visits | 6,002 (4,468) | 6,916 (6,936) | **0.0045** | 3,915 (4,041) | 4,796 (3,867) | 0.24 | 4,057 (3,071) | 6,974 (8,121) | 0.48 |
| Outpatient visits | 768 (550) | 928 (728) | 0.25 | 1,226 (502) | 946 (840) | 0.018 | 959 (843) | 1139 (843) | 0.70 |
| Primary care visits | 926 (1,020) | 757 (1,236) | 0.37 | 429 (385) | 534 (600) | 0.97 | 918 (786) | 765 (822) | 0.58 |
| Mean of all mean visit costs | 3,627 (4,919) | 5,882 (7,153) | 0.49 | 3,466 (3,449) | 2,715 (3,719) | 0.11 | 3,866 (3,546) | 4,999 (7,331) | 0.76 |
| Biologic treatment | 11,208 (8,434) | 5,068 (5,845) | **<0.0001** | 10,648 (8,370) | 6,296 (5,828) | **0.022** | 13,020 (7,022) | 6,695 (9,334) | **<0.0001** |

Data are mean (SD). Costs are calculated in GBP. Abbreviations: CD, Crohn’s disease; IBD, inflammatory bowel disease; UC, ulcerative colitis.
